# Undergoing Cytoreductive Surgery for Colorectal Peritoneal Metastases: A Qualitative Exploration of the Lived Experiences of Patients and Carers

**DOI:** 10.1245/s10434-025-17351-4

**Published:** 2025-04-28

**Authors:** Pratik Raichurkar, Kilian G. M. Brown, Kate White, Cherry E. Koh, Nabila Ansari, Nima Ahmadi, Michael J. Solomon, Daniel Steffens

**Affiliations:** 1 Department of Colorectal Surgery, Royal Prince Alfred Hospital, Sydney, NSW Australia; 2 Surgical Outcomes Research Centre (SOuRCe), Royal Prince Alfred Hospital, Sydney, NSW Australia; 3 Institute of Academic Surgery (IAS), Royal Prince Alfred Hospital, Sydney, NSW Australia; 4 Faculty of Medicine and Health, Central Clinical School, University of Sydney, Sydney, NSW Australia; 5 Sydney Nursing School, University of Sydney, Sydney, NSW Australia; 6 The Daffodil Centre, University of Sydney, A Joint Venture with Cancer Council NSW, Sydney, NSW Australia; 7 Department of Colorectal Surgery, St George Hospital, Kogarah, NSW Australia

**Keywords:** Colorectal cancer, Cytoreductive surgery, Patient experience, Qualitative, Peritoneal metastases

## Abstract

**Background:**

This study explored the lived experience of patients with colorectal peritoneal metastases undergoing cytoreductive surgery and their carers, including the decision to undergo radical surgery, recovery, and long-term survivorship.

**Methods:**

This qualitative study was conducted in a tertiary cytoreductive surgery center. Semi-structured interviews were conducted with an experienced researcher to explore participants’ experiences, challenges, and reflections during different treatment stages. In-depth thematic analysis was performed on interview transcripts using a phenomenological approach to identify key major and minor themes.

**Results:**

Thirteen interviews were conducted, with 5 major themes and 11 minor themes identified*. Understanding diagnosis and treatment implications—*survival was a key driver in decision making, although many did not feel they had a choice or understood the magnitude of the intervention. *Adjustment to uncertainly—*uncertainties regarding surgical intervention, its impacts and long-term recurrence, led to significant burden. *Unmet supportive care needs—*psychology services and patient-led support groups have key roles in addressing long-term support needs. *A positive impact from multidisciplinary care teams—*allied health involvement pre- and postoperatively was associated with a positive experience. *Unexpected long-term physical effects—*unexpected physical effects impacted quality of life during long-term survivorship.

**Conclusions:**

Patients and carers undergoing cytoreductive surgery for colorectal peritoneal metastases endure a difficult journey and decision-making process; an understanding of their lived experience can improve the informed consent process and target supportive care endeavors to areas of need.

Cytoreductive surgery (CRS) for colorectal cancer with peritoneal metastases (CPM) represents a challenge for clinicians and patients alike. In modern rhetoric, selected patients with CPM are eligible for potentially curative surgical resection and intraperitoneal chemotherapy (IPC). As surgical technique improves and our knowledge advances, current trials and data report median survival rates of approximately 40–70 months,^1–3^ providing those with a once palliative prognosis a chance for long-term survival; however, this intervention has significant psychological, emotional, and physical impacts on patients and caregivers alike.^4^ When considering this intervention for patients, clinicians must be aware of the consequences of such radical surgery and the overall patient experience, in order to facilitate informed consent and shared decision making.

To date there has been limited exploration of the experience of patients undergoing CRS for CPM. Current literature in this space focuses on surgical and survival outcomes, with a recent systematic review demonstrating only 2% of studies in this space exploring overall effect on quality of life and patient-reported outcomes.^5^ While surgical, morbidity, and survival outcomes are critical for establishing the safety and oncological efficacy of CRS, the overall experience of patients provides valuable insight into the unique care needs of this population.

This study aimed to explore the lived experience of patients with CPM undergoing CRS (with or without IPC) and their carers, including the decision to undergo such radical treatment, their recovery, and long-term survivorship issues. Utilising an in-depth qualitative analysis of semi-structured interviews, it was hoped the findings would inform perioperative counseling as well as supportive care measures to address areas of unmet need in the future.

## Methods

This phenomenological research study utilized a series of iterative semi-structured interviews with patients who had undergone CRS for CPM and their carers. Data from these interviews were analyzed using Husserl’s methods of interpretive phenomenology^6^ to understand the experience of patients and carers undergoing this treatment. This study is part of a broader research project conducted by the Surgical Outcomes Research Centre (SOuRCe) at the Royal Prince Alfred Hospital (RPAH), which aims to incorporate the views of patients and carers into outcome reporting for CRS and CPM by development of a core outcome set.^7^ This study received ethical approval from the Sydney Local Health District (Protocol No. X23-0318 & 2023/ETH01852).

### Participant Recruitment

Patients undergoing CRS for CPM at the RPAH between April 2017 and December 2023 were identified from a prospectively maintained database. Adult patients who had surgery more than 6 months ago and were fit enough to partake in the interview process (as deemed by their treating physician), and were able to provide informed consent, were eligible to participate. Purposive sampling was used to maintain a representative patient cohort and encourage diversity within the experiences explored. Characteristics including age, primary residence, use of IPC, and presence of a stoma were used to guide recruitment, for which a sampling matrix was published a priori.^7^ Patients were invited to participate through their treating physician and were sent an official invitation, participant information sheet, and written consent form. Interested participants could contact the primary researcher via mail, email, or telephone, or express interest through an online RedCap link. Those interested were contacted by the research team to schedule a 1-h time slot to conduct the interview and were encouraged to ask their carers (if applicable) to participate. Informed consent was obtained through a written consent form, or through a RedCap eConset platform, depending on participant preference.

### Data Collection

Interviews were conducted face-to face at RPAH or via teleconference software (Microsoft Teams) by a single male researcher (PR) who had no prior clinical relation to the participants. The interviewer (PR) is a medical practitioner with clinical experience in managing patients with CPM perioperatively and prior research experience in the CRS field. Experienced qualitative researchers (KW, KB, DS) and clinicians (NA, CK, NAh) supervised the overall interview process. Patients could attend interviews with carers or a support person if they wished. Patient demographics and surgical and perioperative treatment data were extracted from a prospectively maintained database and electronic medical record.

A semi-structured interview guide was developed by a multidisciplinary team of clinicians, researchers, and allied health staff to help facilitate open-ended discussion. This explored the patient’s or carer’s experience with CRS through initial decision making, postoperative experience, and overall reflection on the long-term impacts of treatment. The interview questions and format were refined in an iterative fashion, with the concepts/themes discussed by patients and carers used to inform subsequent interviews. The concept of core outcome sets and the broader context of this study was also explained. Interviews were recorded using digital audio recording software and transcribed by a single researcher (PR), with identifying information removed. Field notes were taken during interviews to assist with identifying themes for the iterative interview process, however they were not part of the formal analysis. Participants were offered an opportunity to review their transcriptions prior to analysis. Interviews continued until data saturation was achieved and no new themes were being discussed.

### Data Analysis

Transcripts were imported into NVivo qualitative analysis software (Nvivo 11, QSR International, Burlington, MA, USA) for review. Thematic analysis was performed using a phenomenological approach to identify meaningful textural (what was experienced) and structural (how it was experienced) elements, which were interpreted to form multiple subjective truths depicting the essence of the patient and carer experience. Interview transcripts were coded by a single researcher (PR) under supervision of an experienced qualitative researcher (KW) and the broader expert team.

Broader themes were generated through discussion between one researcher (PR) and an experienced qualitative researcher (KW) who was not involved in the data collection process. Data and key concepts were assigned codes, and sufficiently similar codes were clustered together through collaborative discussion. Themes were then verified by the broader research team and results were reported in accordance with the Consolidated Criteria for Reporting Qualitative Research (COREQ).^8^

## Results

A total of 13 patients and two carers participated in this study; both carers attended interviews alongside patients. Eleven interviews were conducted via teleconference and two were conducted face-to-face. No interviews were repeated. A summary of demographic information for all patients is presented in Table 1.

**Table 1:** Patient demographics

Characteristic	*N* = 13
Sex	
Male	8 (62)
Female	5 (38)
Age at surgery, years	
40–50	3 (23)
50–60	6 (46)
60+	4 (31)
Primary residence	
Metropolitan	9 (70)
Rural/regional	4 (31)
Type of surgery	
Primary CRS + HIPEC	11 (85)
Re-do CRS + HIPEC	2 (15)
CRS only (no IPC)	0 (0)
HIPEC used	
Oxaliplatin	2 (15)
Mitomycin C	11 (85)
Presence of ostomy	2 (15)
Median time since surgery, months (IQR)	41 (24–52.5)
Mean length of stay, days	15.2
Major complications <30 days (Clavien–Dindo 3+)	2 (15)
Perioperative chemotherapy	13 (100)

Data are expressed as *n* (%) unless otherwise specified

*CRS* cytoreductive surgery, *HIPEC* hyperthermic intraperitoneal chemotherapy, *IPC* intraperitoneal chemotherapy, *IQR* interquartile range

While all patients had CRS over 6 months prior to their interview, individual experiences differed. Three patients had multiple CRS procedures, while some patients did not undergo standard preoperative review by multidisciplinary teams (physiotherapists, psychologists, dieticians, etc.), in part due to the coronavirus disease 2019 (COVID-19) epidemic. A single patient was also enrolled in a prehabilitation trial in which they received increased preoperative multidisciplinary support.

Following the thematic analysis, 5 major themes and 11 minor themes were identified (Table 2).

**Table 2:** Themes and additional quotes

Theme	Subtheme	Quotes
Understanding diagnosis and treatment implications	Survival as the key motivator	“I've still got lots of things I want to do in life. So there was no question, would I, wouldn't I?”
		*“It's (cancer) going to take over me, and it'll probably kill me, but I've got a possibility of another chance.”*
		“I just want to outrun this for as long as possible.”
	A lack of choice	*“It didn't really occur to me that there was another choice, um, I don't think there was.”*
		*“I had no other option, really. It was sort of like, do this or what? There was no ‘and what’?”*
	Understanding the magnitude of intervention	*“Did I understand the severity of the operation and the complexities? I didn't have a clue.”*
		*“I was a little bit dazed by the fact that I had cancer, and I think with (surgeon) it was a similar thing. It was more like, so much information, so much new information.”*
		*“I didn't understand what was going on properly. I felt like I was in good hands, but the anxiety is something that I could have done without.”*
		*“I still didn't comprehend this major surgery, how long it could potentially take and what it entailed.”*
Adjustment to uncertainty	Uncertainty around surgical outcomes	*“We don't know what the outcomes are going to be. But we've got all these good success stories. So you want to go in knowing that there's hope.”*
		*“You'll probably be on chemo for life again. Not great. Might be true, but might not be.”*
		*“Would I get a stoma, would I not? That was quite confronting … you don't know what you don't know.”*
	Unrealistic expectations of recovery	*“It was a little bit scary, because you've always been the sort of person who, is at work or you're doing this, doing that. To see you so incapacitated in a way, it was a little bit scary … I wondered how you're eventually going to bounce back.” (Carer)*
		*“You feel like the goal is to get through the end of surgery, then you realise there's another five years that you've got to get through somehow physically or emotionally.”*
	Burden of recurrence	*“You're just sort of left with this huge burden of anxiety, is it going to come back, is the next CT scan going to show something.”*
		*“Accepting there is a finality to this at some stage and managing it in a positive sense that’s the aim of the game.”*
		*“The psychological side of things is always more, will the cancer come back? Not what impact has the surgery, because for me, the surgery saved my life.”*
		*“I used to write the timing of scans down on the wall to remind me, but the kids would see it. I stopped doing it, so they didn't know what was going on, because they didn't want to know.”*
Unmet supportive care needs	A void of supportive care following the acute phase	*“It's like your life is scheduled by everything that's going to make you better and then suddenly there's nothing. So you feel like you’re in a void in a way.”*
		*“The time after chemo finished was a time when I felt more isolated than any other time. Because I felt like everyone had decided at that point that everything was okay. And so you find yourself somewhere on your own. You don't see the surgeon for another three months or you don't see the oncologist for another three months. I felt very isolated, so I would say that psychologist services or counselling services I think were very important to me.”*
	The potential value of patient-driven support networks	*“… to be able to talk to someone, go and catch up, have a cup of coffee with someone and just ask them, look, did you go through this, you know, did you have these side effects, did you feel this way, how did you handle that? That's something that I would be inclined to engage with because I think that would positive.”*
		*“Being able to go to those groups, and just say, my appointment's tomorrow, I’m having a shocker. Even though they can't physically help, they're not there, they can just say, yeah, know how you feel, and understand.”*
A positive impact from multidisciplinary care teams	Multidisciplinary preoperative interventions have a profound impact	*“I did five weeks, three days a week, or three sessions a week of physical training to get me into shape, which was fantastic. Now, that was so good, because it got me not only physically, but emotionally, mentally and nervous system-wise, ready.”*
		*“When the physio started coming out, I didn't really initially see the point, because, again, I've never been in hospital and I didn't feel like I needed a physio because I'd just been sitting there.”*
		*“… the thoroughness and the total team approach that was taken prior to the operation. It wasn't just between the surgeon and I. All the various meetings with all of the medical and allied health professionals that were involved … That gave me a great deal of confidence that I was being looked after by a tight team.”*
	Long-term benefits of psychology	*“My experience was this overwhelming load of mental and emotional stuff to deal with, but at the same time, if you get help, if you sort of approach it in a certain way, I feel like you can come out of it feeling more comfortable.”*
Unexpected long-term physical effects	Unexpected long-term physical effects	*“I think people's sex life is affected …. I don't think it was expected.”*
		*“I feel like I've become a different person really since that. I don't sort of talk to people, I don't tell people very much about anything. Because no one truly understands.”*
		*“I'm sort of distantly concerned that bowel changes might indicate there's cancer again.”*
		*“I always had it in my head … that I was going to get back to work. There's nothing stopping me.”*

*CT* computed tomography

### Understanding the Diagnosis and Treatment Implications

The majority of participants indicated that survival was their main priority when considering surgery and was the driving force behind accepting such invasive treatment. Patients sighted personal motivations, such as the birth of grandchildren and participation in family milestones, as key motivators in the decision-making process.

*“It's (cancer) going to take over me, and it'll probably kill me, but I've got a possibility of another chance.”*

Another key motivator for some patients was the opportunity to be relieved from chemotherapy, even for a short period of time. This was especially true in patients who had iterative procedures, having gone through multiple cycles of systemic therapy.

However, a common theme among patient decision making was the feeling of having no choice. In some cases, patients were aware of alterative options, while in other cases patients were unaware a choice even existed. Given the only chance of long-term survival was surgical resection, most patients did not feel there was a decision to be made and this pathway was the only one forward. Patients recalled feelings of elation and relief when they were offered surgery, particularly when their eligibility was uncertain at the time of referral. For some patients this led to a sense of obligation to undergo surgery when offered.

*“I had no other option, really. It was sort of like, do this or what? Like, there was no ‘and what?’”*

During preoperative counseling, it is clear the true implications of surgery and the magnitude of the journey ahead was not always appreciated. While patients recalled that the surgery was adequately explained during consultations with their treating specialist with the aid of pamphlets, diagrams, and allied health input, there was variation in the level of understanding. This was especially true in patients who were newly diagnosed with cancer as opposed to those who had previously undergone surgery and/or multiple lines of chemotherapy. This led to uncertainty among participants at the time of referral and difficulty knowing what information was personally important to them and which questions to ask.

*“Did I understand the severity of the operation and the complexities? I didn't have a clue.”*

On the other hand, a positive impact was noted by patients who had surgical management explained by their referring medical oncologist prior to consultation with a peritoneal malignancy surgeon. Patients reflected that this initial counseling allowed them to be more prepared for their consultation and allowed them to reflect upon what information could be important to them prior to making treatment decisions.

### Adjusting to Uncertainty

Managing uncertainty was a central part of the patient experience, through the initial work-up for surgery and into long-term survivorship. Adjusting to this new uncertain state presented difficulties for patients, who in some cases found real benefit from a network of personal supports and psychologists. In the preoperative phase, patients felt uncertainty about the physical implications of the surgery, for example the presence of ostomies and which organs may require resection. There was also uncertainty about which adjuvant treatments may be required, and the prospect of chemotherapy was incredibly daunting to some, even more so than surgery.

*“I was told you'll probably be on chemo for life again. Not great. Might be true, but might not be.”*

This uncertainty continued well into the recovery period, where patients commonly met an unexpectedly drawn-out process. This experience was physically much more taxing and impactful on daily living than anticipated by patients and carers, the length of which challenged patients’ previous understanding of surgical recovery. This was evident in both patients who had no prior surgery for comparison and patients who had prior surgery, the recovery of which was nowhere near as drawn out. Despite being present for the majority of consultations, carers also expressed difficulty in grappling with the immediate consequences of surgery.

*“To see you so incapacitated in a way, it was a little bit scary … I wondered how you're eventually going to bounce back.” (Carer)*

Adjusting to uncertainty became the most difficult as this sensation pervaded into the long-term. It was clear from the interviews that anxiety around an unpredictable disease recurrence pattern and uncertainty during surveillance had a significant impact on a patient’s mental well-being. In these cases, patients sought support from loved ones, psychologists, and patient groups, which were all reported to have positive effects. However, the chronic nature of this state and the resurfacing of anxiety at the time of scans and blood tests remained a great burden to the vast majority of patients and their families/carers. Stemming from this phenomenon, patients reflected on feeling burdensome to family members and, in due course, being alienated from social networks. Carers were not always able to, or did not know how to, provide support in the most effective way during this time.

“*You're just sort of left with this huge burden of anxiety, is it going to come back, is the next CT scan going to show something.”*

### Unmet Supportive Care Needs

Many participants noted that in the short-term postoperative period, consistent follow-up and appointments provided a metaphorical safety net. However, after this acute phase, as these appointments become more sporadic, many patients expressed a void of support, voicing feelings of isolation and anxiety. During this period, some participants gained increasing value from formal psychology services.

*“It's like your life is scheduled by everything that's going to make you better and then suddenly there's nothing. So you feel like you’re in a void in a way.”*

*“The time after chemo finished was a time when I felt more isolated than any other time. Because I felt like everyone had decided at that point that everything was okay. And so you find yourself somewhere on your own … .”*

Some patients identified that a means to improve their experience postoperatively could be through patient-led support networks or similar online forums. Despite the potential value identified, only two patients were able to access such networks, leaning on groups focusing on similar conditions such as colorectal cancer broadly or pseudomyxoma peritonei (PMP). Patients also sought information from dieticians and psychologists, however many patients with a keenness to engage did not know how.

### A Positive Impact from Multidisciplinary Care Teams

Patients who had consultations with allied health staff, physiotherapists, psychologists, stomal therapy, and dieticians preoperatively reported the positive impacts of these sessions. These aided patients in feeling a sense of holistic care, instilling confidence in the treatment course to come, and empowering them to engage with these teams postoperatively. Those who did not receive this same opportunity and briefing took longer to engage in recovery, sometimes finding difficulty in understanding interventions.

*“… the thoroughness and the total team approach that was taken prior to the operation … . All the various meetings with all of the medical and allied health professionals that were involved … . That gave me a great deal of confidence … .”*

Multimodal management strategies also improved the long-term experience, with psychology services being of paramount importance during long-term survivorship. These services also aided in patients managing the aforementioned ongoing uncertainty.

### Unexpected Long-Term Physical Effects

Patients also reported a myriad of long-term sequelae, which, in some cases, were anticipated but often surprising. These included constipation, fecal urgency, fatigue, and sexual dysfunction. A significant variability was noted in patients’ ability to return to work following surgery; while most identified this as a key milestone in their recovery, not all were able to achieve this. This had significant follow-on impacts on their financial situation and sense of independence. Additionally, unpredictable bowel symptoms, including constipation, diarrhea, and urgency, had effects on quality of life as well as the ability to meaningfully engage in social activities. An overarching difficulty for most patients was managing fatigue and lethargy, which may in part be due to adjuvant chemotherapy commencing during their recovery.

These physical symptoms, notably surgical scars and umbilical sacrifices, served as a continuous reminder of their journey. Patients also reported that physical symptoms provoked anxiety as to whether they may be an indicator of a surgical complication or disease recurrence.

*“I'm sort of distantly concerned that bowel changes might indicate there's cancer again.”*

## Discussion

Through a series of iterative interviews, this study provided in-depth exploration of the lived experience of patients and their carers, undergoing CRS from the time of initial work-up through to longer-term challenges. CRS offers a chance at long-term survival for select patients with CPM, however it is associated with a high burden of morbidity.^9^ The key findings of this study are that patients endure an uncertain treatment course and a void of clinical support in a quest to achieve long-term survival. When making the decision to undergo surgery, patients consider survival as their primary goal; however, few feel like there is a choice to weigh up in this regard, and given the prognosis of other ineligible patients, sometimes feel obligated to accept further treatment. Many patients and carers felt that recovery from surgery was significantly more taxing than anticipated and the psychological challenge of managing an uncertain postoperative course immense. It is clear the patients undergoing CRS for CPM face major challenges, which should be navigated and acknowledged by clinicians and allied health professionals. This is especially important as CRS emerges as the standard of care for CPM and maintaining patient-centerd care models.^10^ An acknowledgment of the uncertainties patients face as well as some of the rationale behind accepting treatment can be utilized to improve the informed consent process and preoperative counseling. These considered discussions on the potential physical and psychosocial impacts of CRS may help to pre-empt and mitigate some of the postoperative uncertainty patients and carers face.

Our findings depict a difficult journey for CPM patients, with unique challenges faced at the time of diagnosis, postoperatively and in the longer term. Patients and carers struggled to comprehend the magnitude of the intervention before them, despite in-depth and considered perioperative counseling. This phenomenon has been echoed in similar studies and may partly pertain to the inherently heterogenous nature of the intervention and limitations of preoperative imaging, resulting in many unknowns until the time of surgical exploration.^11,12^ There is a positive role for early multidisciplinary interventions in this setting, with patients reporting both psychological and physical preparedness for surgery when employed. This strengthens positive arguments for multimodal prehabilitation and affirms that the value current literature is demonstrating extends beyond surgical outcomes to the overall patient experience.^13,14^ Moreover, the findings highlight that patients with CPM require tailored interventions to address unique emotional, practical, and physical challenges. This study also demonstrates the benefits of repeated counseling, as education from referring physicians and oncologists allowed patients time to critically evaluate their own concerns. As CRS remains a novel intervention performed in specialized centers, referring clinicians may not be wholly aware of what surgery entails. Clinician education around this is crucial in giving patients time-critical counseling and improving readiness.^15^

It is known that patients undergoing CRS undergo a prolonged recovery, with most studies indicating that 6–12 months are required before symptomatology is manageable and quality of life returns to baseline.^16,17^ Patients continue to experience gastrointestinal symptoms, fatigue, and sleep issues long after this period, and targeted survivorship programs have been proposed by other qualitative studies identifying similar issues.^18,19^ These programs can help to ease adjustment to retirement, mitigate social implications, and assist with feelings of burden to carers and family members. Psychological well-being and the fear of recurrence was a dominating aspect of long-term survivorship. This study found a potential void of clinical support during the chronic stage of disease, ongoing follow-up with psychology services, and referral to local support services would be of great benefit to mental well-being during this period.^13,20^ Targeted carer education could also support patients, as this study found carers did not always know the best way to provide support, especially while grappling with their personal experiences. Such education could also reduce the risk of carer burnout, which has negative implications for patients and carers alike.^21^ Patients commonly yearned for patient-driven support networks or groups through physical meetings or online platforms, which have both shown to improve quality-of-life outcomes in other tumor streams.^22^ Although there are existing groups for patients with PMP and colorectal cancer, there is no current patient network for CPM, and this could be a promising area of innovation in supportive care.

To our knowledge, this is the first study to apply qualitative exploration specifically on CRS patients with CPM. These patients have unique experiences and issues that are unlikely to be captured in generic or colorectal cancer-specific quality-of-life instruments, and a qualitative approach is therefore ideal to explore these. This study is limited by its single-center recruitment; however, through use of a purposive sampling matrix, patients with a mix of experiences, age, and demographic backgrounds were captured. Common themes were also raised by many patients and hence these findings are likely to be relevant to the majority of patients with CPM. However, this study only captured patients who were relatively well and enjoyed long-term survival, and this survivorship bias may have skewed the results. The experience of patients with early recurrence, poorer survival outcomes, and within the immediate postoperative period may not have been captured. Additionally, patients were asked to identify and invite carers to participate, which may present a selection bias. Furthermore, the small number of participating carers limits any firm conclusion about the carer perspective.

## Conclusion

This qualitative analysis provided insight into the complex, lived experience of patients undergoing CRS for CPM. An acknowledgment of this experience by clinicians may assist in the complex preoperative decision-making process, as well as guiding patient-centered perioperative counseling. Future postoperative supportive care endeavors should also be tailored towards this unique patient experience, and particularly focus on long-term psychological support, patient-led survivorship networks, and multimodal prehabilitation.

## References

[1] Quenet F, Elias D, Roca L, et al. Cytoreductive surgery plus hyperthermic intraperitoneal chemotherapy versus cytoreductive surgery alone for colorectal peritoneal metastases (PRODIGE 7): a multicentre, randomised, open-label, phase 3 trial. *Lancet Oncol*. 2021;22(2):256–66. 10.1016/S1470-2045(20)30599-4. 33476595 10.1016/S1470-2045(20)30599-4

[2] Mirnezami R, Mehta AM, Chandrakumaran K, et al. Cytoreductive surgery in combination with hyperthermic intraperitoneal chemotherapy improves survival in patients with colorectal peritoneal metastases compared with systemic chemotherapy alone. *Br J Cancer*. 2014;111(8):1500–8. 10.1038/bjc.2014.419. 25225906 10.1038/bjc.2014.419 PMC4200082

[3] Livin M, Leonard D, Bachmann R, et al. Cytoreductive surgery and hyperthermic intraperitoneal chemotherapy for peritoneal carcinomatosis from colorectal cancer: a 13 years-retrospective monocentric study. *Acta Gastroenterol Belg Oct-Dec*. 2022;85(4):573–9. 10.51821/85.4.10811. 10.51821/85.4.10811 36566366

[4] Chia CS, Tan GH, Lim C, Soo KC, Teo MC. Prospective quality of life study for colorectal cancer patients with peritoneal carcinomatosis undergoing cytoreductive surgery and hyperthermic intraperitoneal chemotherapy. *Ann Surg Oncol*. 2016;23(9):2905–13. 10.1245/s10434-016-5203-6. 27016293 10.1245/s10434-016-5203-6

[5] Raichurkar P, Brown K, Koh C, et al. Reported outcomes following cytoreductive surgery for colorectal peritoneal metastases: a systematic review to inform evidence-based practice and international consensus. *Colorectal Dis*. 2025;27(1):e17280. 10.1111/codi.17280. 39734262 10.1111/codi.17280

[6] Tassone BG. The relevance of Husserl’s phenomenological exploration of interiority to contemporary epistemology. *Palgrave Communications*. 2017;3(1):17066. 10.1057/palcomms.2017.66.

[7] Raichurkar P, Brown K, Ansari N, et al. Developing a core outcome set for cytoreductive surgery for colorectal cancer with peritoneal metastases: a mixed-method study protocol. *Gastrointestinal Disorders*. 2024;6(1):143–51.

[8] Tong A, Sainsbury P, Craig J. Consolidated criteria for reporting qualitative research (COREQ): a 32-item checklist for interviews and focus groups. *International Journal for Quality in Health Care*. 2007;19(6):349–57. 10.1093/intqhc/mzm042. 17872937 10.1093/intqhc/mzm042

[9] Hallam S, Tyler R, Price M, Beggs A, Youssef H. Meta-analysis of prognostic factors for patients with colorectal peritoneal metastasis undergoing cytoreductive surgery and heated intraperitoneal chemotherapy. *BJS Open*. 2019;3(5):585–94. 10.1002/bjs5.50179. 31592510 10.1002/bjs5.50179 PMC6773657

[10] Sedighim S, Khan A, Li AY, et al. Adoption of cytoreductive surgery in the management of peritoneal malignancies-Global trends. *J Surg Oncol*. 2023;128(6):1021–31. 10.1002/jso.27448. 37818906 10.1002/jso.27448

[11] Dobbelaer J, Ceelen W, Pape E, De Meyer S, Cosyns S. CN69 Experiences of patients after cytoreductive surgery and hyperthermic intraperitoneal chemotherapy: A qualitative study. *Annals of Oncology*. 2023;34:S1245. 10.1016/j.annonc.2023.09.1662.

[12] Leo Swenne C, Jangland E, Arakelian E. Patients’ experiences of their everyday life 14 months after cytoreductive surgery and hyperthermic intraperitoneal chemotherapy – a qualitative follow-up study. *Scandinavian Journal of Caring Sciences*. 2017;31(4):904–13. 10.1111/scs.12412. 28124449 10.1111/scs.12412

[13] van Rooijen S, Carli F, Dalton S, et al. Multimodal prehabilitation in colorectal cancer patients to improve functional capacity and reduce postoperative complications: the first international randomized controlled trial for multimodal prehabilitation. *BMC Cancer*. 2019;19(1):98. 10.1186/s12885-018-5232-6. 30670009 10.1186/s12885-018-5232-6 PMC6341758

[14] Minnella EM, Bousquet-Dion G, Awasthi R, Scheede-Bergdahl C, Carli F. Multimodal prehabilitation improves functional capacity before and after colorectal surgery for cancer: a five-year research experience. *Acta Oncologica*. 2017;56(2):295–300. 10.1080/0284186X.2016.1268268. 28079430 10.1080/0284186X.2016.1268268

[15] Ashvin R, Aditi B, Nikhilesh J. Preoperative management of patients undergoing cytoreductive surgery and hyperthermic intraperitoneal chemotherapy. *Indian J Surg Oncol*. 2017;8(4):573–9. 10.1007/s13193-016-0517-1.

[16] Steffens D, Makker PGS, Ansari N, et al. The influence of postoperative morbidity on medium to long-term quality of life trajectories following cytoreductive surgery for peritoneal malignancy: a prospective cohort study. *Surgical Oncology Insight*. 2024;1(1):100004. 10.1016/j.soi.2023.100004.

[17] Wang YF, Wang TY, Liao TT, et al. Quality of life and symptom distress after cytoreductive surgery and hyperthermic intraperitoneal chemotherapy. *World J Clin Cases*. 2022;10(32):11775–88. 10.12998/wjcc.v10.i32.11775. 36405273 10.12998/wjcc.v10.i32.11775 PMC9669838

[18] Falla-Zuniga LF, King MC, Pawlikowski K, et al. Quality of life after cytoreductive surgery and hyperthermic intraperitoneal chemotherapy (CRS/HIPEC): cancer survivors’ perspective through in-depth interviews. *Ann Surg Oncol*. 2024;31:7122–32. 10.1245/s10434-024-15719-6. 39060692 10.1245/s10434-024-15719-6

[19] Francescutti VA, Maciver AH, Stewart E, et al. Characterizing the patient experience of CS/HIPEC through in-depth interviews with patients: identification of key concepts in the development of a patient-centered program. *Ann Surg Oncol*. 2019;26(4):1063–70. 10.1245/s10434-018-07120-x. 30603814 10.1245/s10434-018-07120-x

[20] Joshi A, Larkins S, Evans R, Moodley N, Brown A, Sabesan S. Use and impact of breast cancer survivorship care plans: a systematic review. *Breast Cancer*. 2021;28(6):1292–317. 10.1007/s12282-021-01267-4. 34146242 10.1007/s12282-021-01267-4

[21] Stenberg U, Cvancarova M, Ekstedt M, Olsson M, Ruland C. Family caregivers of cancer patients: perceived burden and symptoms during the early phases of cancer treatment. *Soc Work Health Care*. 2014;53(3):289–309. 10.1080/00981389.2013.873518. 24628120 10.1080/00981389.2013.873518

[22] Kiemen A, Czornik M, Weis J. How effective is peer-to-peer support in cancer patients and survivors? A systematic review. *J Cancer Res Clin Oncol*. 2023;149(11):9461–85. 10.1007/s00432-023-04753-8. 37120782 10.1007/s00432-023-04753-8 PMC10374798

